# A comprehensive genomic and transcriptomic dataset of triple-negative breast cancers

**DOI:** 10.1038/s41597-022-01681-z

**Published:** 2022-09-24

**Authors:** Qingwang Chen, Yaqing Liu, Yuechen Gao, Ruolan Zhang, Wanwan Hou, Zehui Cao, Yi-Zhou Jiang, Yuanting Zheng, Leming Shi, Ding Ma, Jingcheng Yang, Zhi-Ming Shao, Ying Yu

**Affiliations:** 1 State Key Laboratory of Genetic Engineering, School of Life Sciences and Human Phenome Institute, Fudan University, Shanghai, China; 2 Key Laboratory of Breast Cancer in Shanghai, Department of Breast Surgery, Precision Cancer Medicine Center, Fudan University Shanghai Cancer Center, Shanghai, China; 3 Shanghai Cancer Hospital/Cancer Institute, Fudan University, Shanghai, 200438 China; 4 Fudan-Gospel Joint Research Center for Precision Medicine, Fudan University, Shanghai, 200438 China; 5 Greater Bay Area Institute of Precision Medicine, Guangzhou, Guangdong China

**Keywords:** Breast cancer, Cancer genomics

## Abstract

Molecular subtyping of triple-negative breast cancer (TNBC) is essential for understanding the mechanisms and discovering actionable targets of this highly heterogeneous type of breast cancer. We previously performed a large single-center and multiomics study consisting of genomics, transcriptomics, and clinical information from 465 patients with primary TNBC. To facilitate reusing this unique dataset, we provided a detailed description of the dataset with special attention to data quality in this study. The multiomics data were generally of high quality, but a few sequencing data had quality issues and should be noted in subsequent data reuse. Furthermore, we reconduct data analyses with updated pipelines and the updated version of the human reference genome from hg19 to hg38. The updated profiles were in good concordance with those previously published in terms of gene quantification, variant calling, and copy number alteration. Additionally, we developed a user-friendly web-based database for convenient access and interactive exploration of the dataset. Our work will facilitate reusing the dataset, maximize the values of data and further accelerate cancer research.

## Abstract (summary)

**Table:** 

Measurement(s)	RNA expression profiling • whole-exome sequencing (WES) • somatic mutations • copy number alterations (CNAs)
Technology Type(s)	RNA sequencing • DNA sequencing • OncoScan CNV assay
Factor Type(s)	Intervention or procedure
Sample Characteristic - Organism	Homo sapiens
Sample Characteristic - Location	China

## Background & Summary

Triple-negative breast cancer (TNBC) is the most aggressive form of breast cancer and is characterized by the lack of expression of estrogen receptor (ER) and progesterone receptor (PR), and the lack of amplification of the human epidermal growth receptor 2 (HER2)^1,2^. This feature leads to the ineffectiveness of the main targeted therapeutic treatments for TNBC patients. Meanwhile, the high heterogeneity of TNBC makes it possible for each subtype to find its specific etiology and optimal treatment, suggesting the requirement of precise patient subtyping^3–5^. Therefore, molecular profiling-based subtyping is an important strategy for the study of TNBC to achieve precise drug administration^6–9^.

To provide a broader molecular profile of TNBC, we performed a large single-center multiomics study of TNBC at the Fudan University Shanghai Cancer Center (FUSCC)^10^. A unique dataset was established including gene quantification, variant calling, and copy number alteration (CNA) data of 465 primary Chinese TNBCs. This research helped discover actionable targets for different subtypes and explore connections of multiomics features with clinical information, especially in East Asian TNBC patients^11,12^. Since its initial publication in 2019, this dataset has been utilized extensively by the scientific community, deepening the understanding of the heterogeneity about the immune microenvironment in TNBC, facilitating the discovery of multiple molecular biomarkers, and the development of prognostic models for TNBC patients^13–27^.

However, most of the follow-up studies utilized only a part of the rich dataset, suggesting that the full potential of the dataset has not been realized, presumably due to a lack of detailed description and user-friendly toolsets to access the datasets. Specifically, the dataset has not been sufficiently described and details of the analysis pipelines were not fully available in our previous article and other follow-up studies. Moreover, there have been updates of mainstream databases (e.g. the adoption of the human reference genome hg38 over hg19) and software packages previously used. Importantly, the reproducibility of results with the updated pipelines has not been evaluated. Therefore, a detailed description of the dataset with special attention to data quality as well as reanalyzing this dataset using the updated pipelines are urgently needed for facilitating reusing this TNBC dataset.

**Fig. 1:**
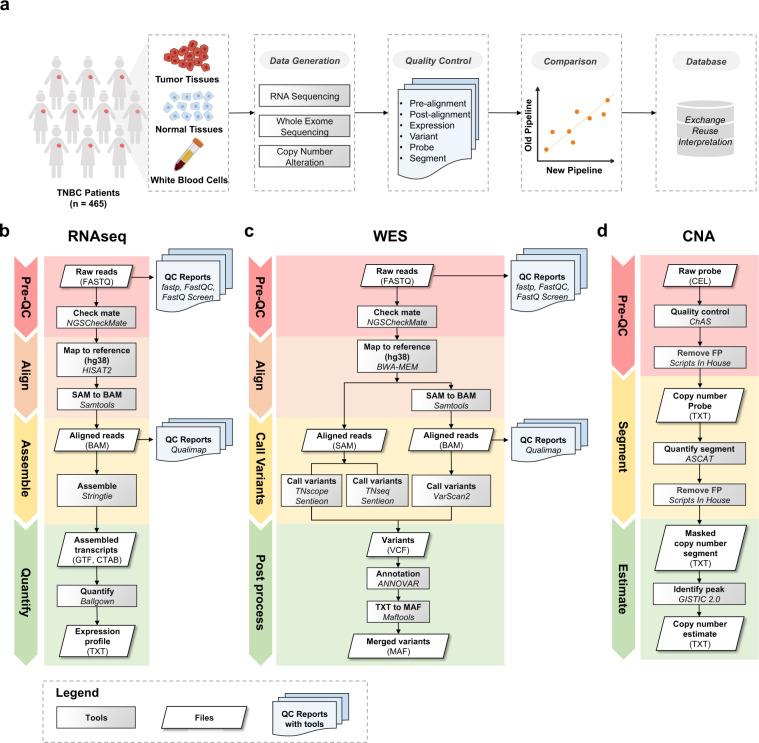
Schematic overview of the multiomics TNBC dataset. (**a**) Data generation and data processing. Detailed workflows of data processing pipelines including (**b**) RNAseq pipeline, (**c**) WES pipeline, and (**d**) CNA pipeline. The tools/algorithms, QC reports, and files are marked with grey, blue and white backgrounds, respectively. FP: False Positive.

In this Data Descriptor, we performed various rigorous quality control (QC) to ensure the data quality from RNA sequencing (RNAseq), whole-exome sequencing (WES), and OncoScan copy number variations (CNV) assay. Additionally, we updated the dataset with a detailed description of the processing steps. By comparing the updated and previously published results, we found good concordance of gene quantification, variant calling, and CNA, demonstrating the excellent reproducibility of the analysis results and pipelines. Eventually, we stored the updated profiling files in the data portal (http://fudan-pgx.3steps.cn/cdataportal/study/summary?id=FUSCC_BRCA_2022) to help with data exchange, interpretation, and reuse. The updated dataset will allow researchers to better conduct TNBC studies, thereby facilitating the development of precision medicine in breast cancer. A visual summary of the study design and workflow is shown in Fig. 1. In addition, the number of samples associated with each omics data type can be seen in Table 1.

**Table 1:** Constitution of a multiomics dataset of TNBC cohort.

Omics	Number of patients with matched samples (n)		Number of patients with tumor samples only (n)	Total number of patients (n)
	Matched tumor tissues and normal tissues	Matched tumor tissues and white blood cells		
Transcriptome (RNAseq)	88		272	360
Genome (WES)		279		279
Genome (CNA)		23	378	401

## Methods

The following is a modified version of Methods previously described in Yi-Zhou Jiang *et al*.^10^.

### Sample collection and preparation

A total of 504 consecutive Chinese patients diagnosed with malignant breast cancer were retrospectively selected for our TNBC cohort. All human research included in the present study was approved by the FUSCC Ethics Committee, and each patient provided written informed consent.

We removed poor-quality samples that were defined as those with none qualified omics data and generated the final cohort of 465 TNBC patients for further analysis. The tumor tissues were freshly frozen with a higher than 50% tumor cell percentage and a stromal cell content lower than 30%. Matched white blood cells and normal tissues were collected for DNA and RNA extraction, respectively. The multiomics dataset includes RNAseq data of 360 patients, WES data of 279 patients, and CNV array data of 401 patients (Table 1).

### RNA isolation and RNAseq

Total RNA from tissues previously stored in RNA-later solution was purified by a MiRNeasy mini kit (Qiagen, Hilden, Germany). RNA library preparation was performed from 0.3–1 µg of total RNA as described in the Illumina TruSeq Stranded Total RNA LT sample preparation kit with Ribo-Zero Gold (Illumina Inc., San Diego, CA, USA). All processes involved were implemented according to the manufacturers’ manuals. After using an Agilent 2100 Bioanalyzer (Agilent Technologies) with the DNA chip to test quality and using a Qubit® 3.0 fluorometer (Invitrogen, Carlsbad, CA, USA) to conduct further quantification, the libraries were sequenced on the Illumina HiSeq platform (Illumina Inc., San Diego, CA, USA), and 150 bp paired-end reads were generated as FASTQ files. For each library preparation from tissue, 12 samples were loaded in a single lane. Additional batch information was also collected for the subsequent elimination of batch effects.

### Data processing and quality control of RNAseq

Preliminary processing of raw FASTQ reads was performed using fastp v0.19.6^28^ to remove adapter sequences. In the process, fastp v0.19.6^28^ was used to check the quality of reads before trimming and FastQC v0.11.5 (https://www.bioinformatics.babraham.ac.uk/projects/fastqc/) after trimming. Then, we used FastQ Screen v0.12.0^29^ to extract the first 10,000 reads from the clean FASTQ files to detect whether the raw data were contaminated with other species, junction primers, etc. Qualimap v2.0.0^30^ was used to calculate the quality of the mapping process. We randomly selected 10% bam files for quality testing for efficiency and cost-effectiveness purposes.

We conducted read alignment and quantification using HISAT v2.1^31^, SAMtools v1.3.1^32^, StringTie v1.3.4^33^ and Ballgown v2.14.149^34^. Genome Reference Consortium human genome build 38 (Genome version: GRCh38_snp_tran) and gene models from Ensembl (version: Homo_sapiens.GRCh38.93.gtf) were used for read mapping and gene quantification. Also, to remove batch effects and other unwanted variations in the dataset, we applied the RUVg method of the RUVSeq package^35^ to preprocess raw read count data. The RUVg read counts were used for sex check and differential expression analyses. Moreover, transcripts per kilobase of exon model per million mapped reads (TPM) were used to normalize the gene expression. To choose genes with accurate expression values, we removed genes with TPM values equivalent to zero in more than 30% of samples and applied the log2 ratio to TPM values prior to subsequent analyses. To avoid infinite values, a value of 0.01 was added to the TPM value of each gene before the log2 ratio. TPM values were used to perform principal component analysis (PCA).

Detailed parameters of data processing and quality control pipelines of the RNAseq can be obtained on GitHub (https://github.com/fudan-tnbc/rnaseq-qc-directional).

### Selection of sex-specific genes

Sex-specific genes were identified using transcriptomic expression profiles from GTEx v7.0^36^, consisting of 11,688 samples across 53 non-diseased tissue sites from 714 donors. Differential gene expression (DGE) analyses were conducted between female and male samples in each of 22 tissue types that were not sex-specific tissues. A gene was identified as sex-specific when satisfying the criteria of Student’s t-test *p* < 0.05 and fold change ≥2 or ≤0.5. Sex-specific genes were then identified and used for subsequent sex check, including five male-specific genes (*RPS4Y1*, *DDX3Y*, *EIF1AY*, *KDM5D*, *TXLNGY*) and two female-specific genes (*XIST* and *TSIX*).

PCA was conducted with the univariance scaling, using the prcomp function of R v4.1.2 (https://www.r-project.org, R development core team). Hierarchical clustering analysis (HCA) was performed using Ward linkage based on a distance matrix using the Euclidean method to measure the distance of the samples and genes, using R package pheatmap v1.0.12.

### DNA preparation and WES

Total DNA was isolated from fresh frozen TNBC samples using TGuide M24 (Tiangen, Beijing, China). The purity and quantity of total DNA were estimated by measuring the absorbance at 260 nm (A260) and 280 nm (A280) using a NanoDrop 2000 spectrophotometer (Thermo Scientific, Wilmington, DE, USA). The extracted DNA was considered to be of good quality and suitable for subsequent experiments if the A260/A280 ratio was within the range 1.6–1.9.

Qualified genomic DNA from tumor tissues and matched white blood cell (normal) samples were prepared for WES. A total of 300 ng of each DNA sample fragmented on a Bioruptor Plus sonication system was used to perform end repair, A-tailing, and adapter aligation with an Agilent SureSelectXT Library Prep Kit (Agilent Technologies, Santa Clara, CA, USA) according to the manufacturer’s protocol. An approximate 750 ng of prepared DNA in a volume of 3.4 μL was then captured using Agilent SureSelect Human All Exon V6 (Agilent Technologies) probes, followed by the amplification of the captured library with indexing primers. Quality control was performed using the Agilent 2100 Bioanalyzer (Agilent Technologies) with a DNA chip. After being quantified with a Qubit^®^ 3.0 fluorometer (Invitrogen, Carlsbad, CA, USA), the libraries were sequenced on an Illumina HiSeq platform (Illumina Inc., San Diego, CA, USA), and 150 bp paired-end reads were generated as FASTQ files. For each library preparation from tissue, 12 samples were loaded in a single lane. And, for each library preparation from blood, 20 samples were loaded in a single lane. Finally, we obtained a WES dataset of 279 matched tumor/normal pairs of samples. The batch information was also collected to differentiate the QC metrics of samples.

### Data processing and quality control of WES data

NGSCheckMate^37^ was used to identify whether the raw FASTQ files were from the same individual. To ensure the accuracy of the WES data matching in the TNBC cohort, we performed a pairwise analysis of all WES data using a modified version of NGSCheckMate v1.0.0 software^37^. We first calculated the variant allele frequency (VAF) of approximately 10,000 inherent SNPs in each input FASTQ file to obtain a VAF file. Then, the correlation scores between all VAF files were calculated. The presence of mislabeling was determined based on whether the WES data associated with the same patient ID had significantly higher correlations than the other pairs.

The quality of WES data was assessed using FastQ Screen^29^, FastQC and Qualimap^30^. After the QC process, the WES reads were aligned using BWA-mem^38^ and the BAM files were generated using Samtools^32^. Next, the BAM files were further preprocessed with the Sentieon Genomics tools version 202010.02^39^, which sequentially included (1) duplicates marking; (2) calculating data quality metrics; (3) conducting base quality score recalibration (BQSR); and (4) performing variant calling with TNseq and TNscope.

Three callers were used to identify the somatic mutations, including VarScan2^40^, TNseq^39^, and TNscope^39^. Specifically, for the raw VarScan2 results, processSomatic and somaticFilter were used to extract high-confidence somatic mutations and to remove clusters of false positives and single nucleotide variants (SNV) calls near indels. For the other two callers, a filtering procedure based on a panel of normal (PoN) samples was used to screen out expected germline variation and artifacts. This PoN panel was based on 279 normal samples, from which each VCF file was created corresponding to the sites identified as mutations by TNseq and TNscope, respectively. In addition, the location of the population germline resource containing the population allele frequencies obtained from gnomAD^41^ was used to filter the raw TNseq results.

To obtain the final set of mutation calls, we also used a two-step approach as in the previous publication^10^. First, we removed any spurious variant calls arising as a consequence of sequencing artifacts and then made use of consensus mutations in at least two out of three callers to identify somatic mutations (referred to as “1^st^ Filter Mutation” in subsequent analysis). Secondly, additional filtering based on Bam-readcount^42^ was performed to reduce false positive calls: (1) variant allele frequency (VAF) ≥ 8%; (2) sequencing depth in the region ≥ 8; (3) sequence reads in support of the variant call ≥ 2. Only variants with the following functional classification were considered in this study, i.e, missense mutation, nonsense mutation, nonstop mutation, RNA mutation, silent mutation, variants at splice site or translation start site, insertion and deletion (referred to as “Final Mutation”).

The above process has been packaged into integrated pipelines which can be accessed at https://github.com/fudan-tnbc/. The different analysis phases are encapsulated in separate repositories, including *ngscheckmate_fastq* and *vaf_ncm* for paired assays, *cbcga-wes-qc* and *bam-readcount* for quality control, and *variant-calling* for the detection of somatic mutations. The detailed parameters can also be accessed.

### CNA data generation

Genome-wide copy number analysis was performed using an OncoScan CNV Assay Kit (Affymetrix, Santa Clara, CA, USA) according to the manufacturer’s recommendations. The arrays were washed and stained using a GeneChip Fluidics Station 450 (Affymetrix, Santa Clara, CA, USA) and were scanned using a GeneChip Scanner 3000 7 G (Affymetrix, Santa Clara, CA, USA). The fluorescence of clusters was measured to generate a DAT file. Cluster intensity values were automatically calculated using a built-in algorithm from DAT files using the GeneChip Command Console software (Affymetrix, Santa Clara, CA, USA), and a CEL file was generated. Apart from the 401 tumor samples, we also added 23 randomly selected white blood cell samples from these patients as a reference cohort of DNA to assess the level of recurrent germline/potential false-positive calls.

### Data processing and quality control of CNA

Chromosomal Analysis Suite (ChAS) v4.3 software (Affymetrix) was used to perform quality control of Affymetrix OncoScan CNV SNP probe assays. Common QC metrics for OncoScan CNV data were collected, including the Median of the Absolute values of all Pairwise Differences (MAPD), Normal Diploid Waviness Standard Deviation (ndWavinessSD), and SNP Quality Control of Normal Diploid Markers (ndSNPQC). MAPD and ndWavinessSD represent short-range and long-range noise levels, respectively, and these values are negatively related to the quality of CNA estimation^43^. ndSNPQC measures how well genotype alleles are resolved in the microarray data but are only applicable to normal diploid markers. Higher ndSNPQC values indicate better identification of each genotype.

Moreover, we used R software to pick regions altered in 12 or more of the 23 WBC samples. These regions were doubled in range and were defined as recurrent germline/potential false-positive calls for subsequent removal. The probe-level output from the ChAS v4.3 software was filtered by the log2 ratio of segment-level output. The filtered probe-level file was then analyzed using ASCAT v2.4.3 to obtain segmented copy number calls. These segments from ASCAT v2.4.3 that overlap with previously described recurrent germline/potential false-positive calls were removed.

The filtered segments were subsequently used to produce log2 ratios by dividing the total copy number (nAraw + nBraw, with zero values set to 0.05). These segments were used as the input of GISTIC2.0 v2.0.23^44^ that was run with the same parameter settings as the previous publication^10^ (-ta 0.2 -td 0.2 -genegistic 1 -smallmem 1 -broad 1 -conf 0.95 -rx 0 –brlen 0.7 -cap 3.5 –armpeel 1 -savegene 1).

The files “all_lesions.conf_95.txt” and “all_thresholded.by_genes.txt” were collected from the results of GISTIC2.0^44^ for further analysis. The former file provided CNA information on chromosome peaks and the latter provides the CNA profile of genes. We regarded the old and new different peaks as the same when the following conditions were met (1) both peaks are identical (2) both peaks contain the most identical genes (3) the band range of different peaks fluctuates within 1. Then, we used the Jaccard Index, which represented the number of observations in both sets divided by the number in either set (intersection set divided by union set), to measure the consistency of gene CNA profiles across different pipelines.

### Classification of four TNBC subtypes

The expression profiles of 2,000 protein-coding genes which had the highest SDs (Standard Deviation) in tumor samples from the old expression matrix (Fragments Per Kilobase of exon model per Million mapped fragments (FPKM)) were selected to perform k-means clustering with maximum 1,000 iterations (the “kmeans” function in R, k = 4) in the old analysis. Four subgroups corresponding to the four subtypes of FUSCC TNBC could be obtained, including a luminal androgen receptor (LAR) subtype, an immunomodulatory (IM) subtype, a basal-like and immune-suppressed (BLIS) subtype, and a mesenchymal-like (MES) subtype. The details could be seen in the previous publication^10^. For determining the impact of the new analysis on the molecular subtyping results, we selected the unfiltered FPKM expression profiles and the SD top 2000 genes (only 1889 genes were consistent with the old pipeline because of the shifted genome version) used in the old analysis to subtype classification.

## Data Records

All the omics data and attached metadata can be viewed in the National Omics Data Encyclopedia (NODE, http://www.biosino.org/node) by pasting the accession (OEP000155)^45^. OncoScan CNV data and sequence data have also been deposited in the NCBI Gene Expression Omnibus (OncoScan array; GEO: GSE118527)^46^ and Sequence Read Archive (WES and RNAseq; SRA: SRP157974)^47^. The updated profiling files can also be seen at Figshare (10.6084/m9.figshare.19783498.v5)^48^ in parallel to the Data Portal (http://fudan-pgx.3steps.cn/cdataportal/study/summary?id=FUSCC_BRCA_2022).

## Technical Validation

### Omics data quality control

#### Transcriptomic data QC

**Fig. 2:**
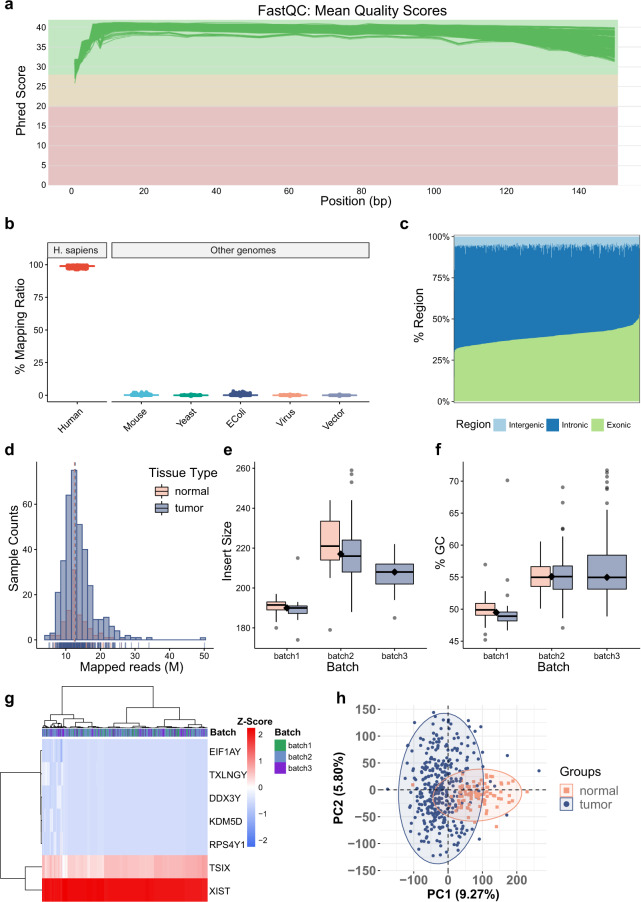
Quality control metrics of RNA-seq data (N = 448). (**a**) Line plot presenting an overview of high-quality scores across all bases at each position in the FASTQ file of all samples. (**b**) Box plot representing mapping ratio (%) across all samples of multiple sources. (**c**) Bar plot representing the percentage of reads that map to gene sequence categories across all samples. (**d**) Frequency distribution plot representing mapped reads. Box plots representing (**e**) median insert size of the data of all samples per batch and (**f**) GC content across all samples per batch (Orange represents the normal group, and purple represents the tumor group). (**g**) Heatmap and hierarchical clustering representing the expression level of the seven sex-specific genes across all samples, including five male-specific genes (RPS4Y1, DDX3Y, EIF1AY, KDM5D, TXLNGY) and two female-specific genes (XIST and TSIX). (**h**) Principal Component Analysis (PCA) of all tumor and normal samples.

**Fig. 3:**
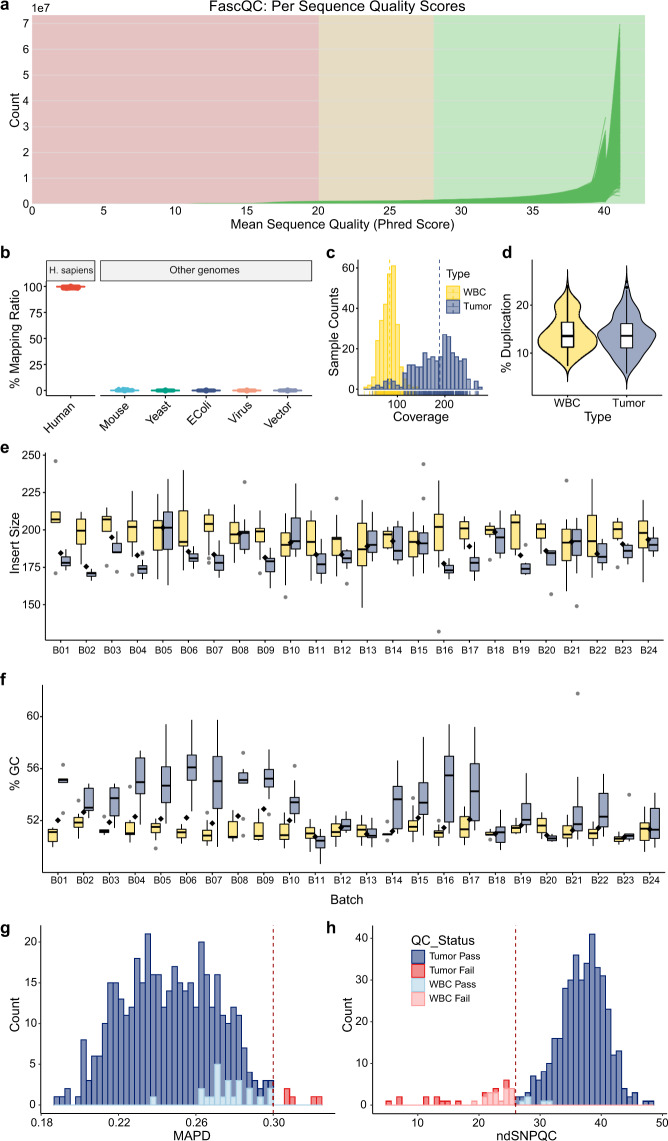
Quality assessment results of genomics data. (**a**) Line plot from FastQC presenting sequences of all samples have universally high quality in WES. (**b**) Box plots representing mapping ratio (%) across all samples of the human genome (left) and of multiple sources (right). (**c**) Frequency distribution plots representing the median coverage of all samples. (**d**) Violin plot representing duplication across all samples. And box plots representing (**e**) median insert size and (**f**) GC content of the data of all samples per batch (Yellow represents the samples from white blood cells and purple represents samples from tumor tissues). Frequency distribution plots representing quality assessment results of OncoScan CNV data, including (**g**) MADP score and (**h**) ndSNPQC score.

**Fig. 4:**
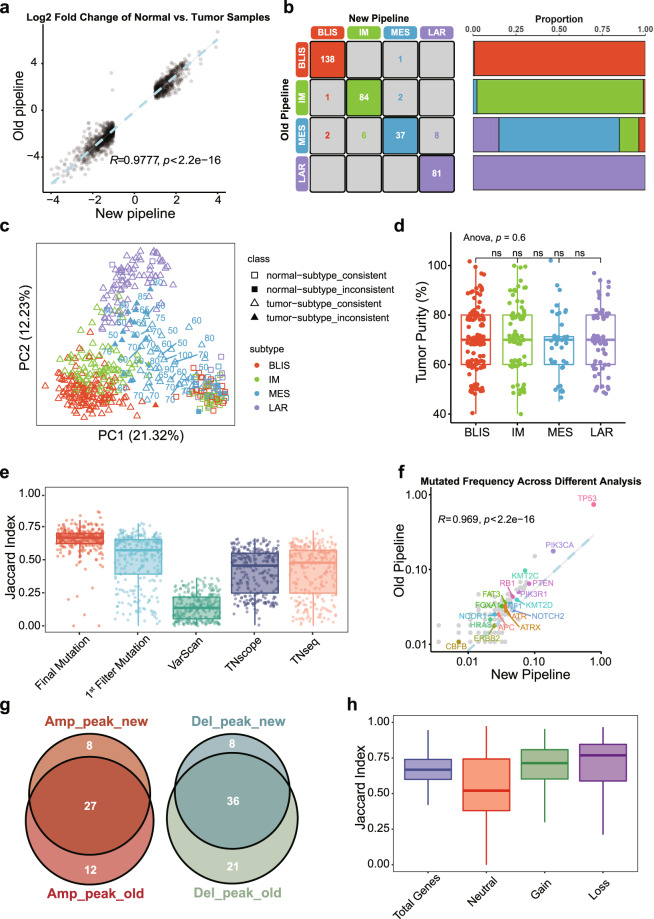
Comparisons of the results from the same dataset but the different pipelines (old vs. new). (**a**) Scatter plot representing consistency of the log2FC of differentially expressed genes by comparing tumor and normal samples across different pipelines based on RNAseq read counts. (**b**) Expression-based (FPKM) unsupervised clustering shows good concordance in TNBC molecular subtyping. BLIS: basal-like and immune-suppressed subtype, IM: immunomodulatory subtype, MES: mesenchymal-like subtype, and LAR: luminal androgen receptor subtype. (**c**) PCA of 360 TNBC patients including 448 samples (360 tumor samples and 88 normal samples) using SD top 2000 genes used in the old analysis. Tumor purity (%) of 40 tumor samples from MES patients (N = 53) was labeled on the graph. Box plots representing (**d**) tumor purity of 315 tumor samples across four molecular subtypes in the study (ns: no significance) and (**e**) the Jaccard index of detected mutations from five mutation datasets through different processes. (**f**) Scatter plot representing the high consistency in allele frequency of all mutated genes between different pipelines (The displays of the x-axis and y-axis are scaled by log10, and the known cancer-related genes were labeled. There are many genes are overlaid on each other in the panel because their mutation frequencies are consistently low.). (**g**) Venn diagrams representing the good consistency at the peak level and the box plot representing (**h**) the Jaccard Index of detected genes across different CNA types.

We conducted quality control of RNAseq data (Fig. 1b) and generated a series of QC metrics RNAseq (Supplementary Table 2). The overall quality of the RNAseq dataset was satisfied at the level of raw and mapped data in the following aspects: (1) the Phred quality scores across all bases at each position in the FASTQ file of all samples were consistently high (Fig. 2a); (2) the mapping ratio of all samples was high (>96%), and no obvious contamination from other sources was observed (Fig. 2b); (3) the distribution of gene region occupancy was consistent with the characteristics of the Ribo-Zero libraries^49^ (Fig. 2c); (4) the average mapped reads of all samples were around 13 Million (Fig. 2d**)**; (5) the average length of insert size was approximately 200 bp (Fig. 2e); (6) the average GC content of the data generated from all samples was around 52% (Fig. 2f).

Besides, we also performed quality control at the expression profile level, including sex check and principal component analysis (PCA) clustering based on tumor and paired normal tissue classification. For sex check, we used seven sex-specific genes, including five male-specific genes (*RPS4Y1*, *DDX3Y*, *EIF1AY*, *KDM5D*, *TXLNGY*) and two female-specific genes (*XIST* and *TSIX*). All samples passed the sex check because they were all from female patients with high expression of female-specific genes and little expression of male-specific genes was observed in our results (Fig. 2g). The PCA results showed that tumor samples and paired normal samples can be well differentiated at the first principal component (Fig. 2h), indicating good quality of RNAseq data at the expression level.

#### Genomic data QC

We performed a pairwise analysis of all WES to ensure accuracy in terms of data matching in the TNBC cohort. Information from RNAseq was added only to assist in determining whether the WES samples were paired or not. The results showed that 98.92% (276/279) of patients were confirmed to have matched tumor-normal samples. Two tumor samples (FUSCCTNBC044, FUSCCTNBC140) did not match the labeled paired samples, and they were not matched with any samples in the cohort. In addition, one tumor sample (FUSCCTNBC030) failed to match the labeled paired sample and matched well with another tumor sample (FUSCCTNBC032) in the cohort. Overall, sample ID mismatch problems occurred in the WES data of three samples, which were labeled as FUSCCTNBC044_WBC, FUSCCTNBC140_WBC, and FUSCCTNBC030_TT. We recommend that researchers remove them in subsequent studies. Detailed information on sample mismatch can be found in Supplementary Table 1.

We then ensured the reliability of the WES data by calculating the key quality metrics in both the raw data and the mapped data (Fig. 1c). The overall quality of the sequence data was satisfied in the following aspects: (1) FastQC results showed a high-quality score per sequence (Fig. 3a); (2) the mapping ratio was higher than 97% for human, and there was no obvious contamination from other sources (Fig. 3b); (3) sequencing coverage was around 50X–250X for tumor samples and 0X-150X for white blood cells, as expected (Fig. 3c); (4) the average duplication rate was 14% (Fig. 3d); (5) average insert size was about 186 ± 13.6 bp for tumor samples while 195 ± 15.7 bp for white blood cells (Fig. 3e), (5) the average GC content was between 50 and 55% (Fig. 3f). More QC metrics results were organized in Supplementary Table 3.

We finally conducted quality control of CNA data (Fig. 1d**)** and generated a series of QC metrics (Supplementary Table 4). The Oncoscan CNV dataset also showed generally good quality. Approximately 91% of samples can pass strict quality control (beyond the thresholds of MAPD and ndSNPQC metrics). However, five samples failed the MAPD metric (with value > 0.3, Fig. 3g), which measured differences between adjacent probes, indicating short-range noise in the microarray data. Data that failed this metric may have too much noise to provide reliable copy number calls. 7% (32/424) samples failed the ndSNPQC metric (with value < 26, Fig. 3h), indicating a possible impact on allele-specific copy number detection or specific SNP calling. We did not exclude any of these failed samples in our Oncoscan CNV dataset, but it was advised to check and consider the data quality with such QC metrics in specific analysis applications.

### Good consistency across different analysis pipelines

Many changes have occurred in the data analysis pipelines after the publication of the FUSCC TNBC article^10^, including but not limited to the shift of the reference genome version from hg19 to hg38, the upgrade of the software version and the change of the analysis tools, etc. To determine the impact of the old (adopted in the previous analysis^10^) and new (adopted in the current analysis) pipelines on the results, we compared the profiling results generated about five years apart by two versions of the pipelines of the FUSCC TNBC dataset.

#### Reanalysis of transcriptomic data

After upgrading the analysis pipeline, we calculated log2 fold change (log2FC) and p-value (*p*) by comparing tumor samples with normal samples in the RNAseq expression profiles (read counts) of the old and new analyses through limma software^50^. The results showed a great consistency rate (Pearson’s *R* = 0.98, *p* < 2.2 × 10^−16^) of these differentially expressed genes (|log2FC| ≥ 1, *p* < 0.05) in the two analyses (Fig. 4a), proving that the updated pipeline has little impacts on the final results. Besides, we performed unsupervised clustering based on the expression profiles obtained from the new pipeline and compared subtypes with the previous clustering results. The clustering consistency rate was found to be high except for the MES subtype (Fig. 4b).

We performed PCA for all tumor and normal samples of 360 patients to determine the potential reason for the inconsistency of the MES clustering. The results showed the inconsistent MES samples were grouped close to other molecular subtype clusters, while a substantial portion of MES tumors was grouped with normal tissues (Fig. 4c). We conducted the H&E analysis to obtain tumor purity, including 315 of 360 tumor samples in the study (Supplementary Table 5). The tumor purity in the four molecular subgroups was not statistically significant (Fig. 4d). Moreover, the range of purity in the MES tumors grouped with normal tissues (45% to 90%) was similar to those grouped with other tumor subtypes (50% to 100%) (Fig. 4c), indicating that normal contamination wasn’t the factor contributed to inconsistent clustering of MES subtypes.

#### Reanalysis of genomic data

Three callers were used in the process of somatic variant calling. Thus, we compared the consistency of variant genes obtained by these three callers between the two pipelines. We performed a two-step approach^10^ which has been mentioned before, defined the mutations that appear in at least two caller results as a filtered set of mutations (1^st^ Filter Mutation), and the final set of mutation calls (Final Mutation). For each mutation set, we calculated the concordance of mutated genes annotated in the old and new results for each patient separately and represented the consistency rate using the Jaccard Index. The analysis showed that Final Mutation exhibited the highest consistency, followed by 1^st^ Filter Mutation. For individual variant callers, the concordance of TNseq, TNscope, and VarScan gradually decreased, with the Jaccard Index even below 0.25 for VarScan. In this case, the performance of the two filtered mutation sets was significantly affected by VarScan (Fig. 4e). The above results demonstrated the superiority of the strategy of integrating multiple callers for variant calling. Variants detected by a single caller may include more false positives that cannot be completely excluded based on hard filtering criteria. To further ensure the reliability of the results, we compared the mutation frequencies of well-known cancer-related genes and the results still showed a high concordance (Fig. 4f).

Furthermore, we reanalyzed CNA status using the updated pipeline and compared the concordance of CNA amplification and deletion peaks in different pipelines. The results showed moderate consistency (Jaccard Index was 0.57 and 0.55, respectively) at the peak level^51^ (Fig. 4g). The reduction of total CNA numbers may be due to the stricter threshold when eliminating false positive positions and the updated version of GISTISC2.0 as well as the utilization of the hg38 genome. We listed the CNA results and related genes and highlighted different loci between the old and new analyses (Supplementary Table 6). Moreover, we analyzed the consistency of different CNA types (neutral, gain, loss, and total) genes in different pipelines based on genes that were detected in both pipelines. It showed satisfying consistency of CNA types, including gain, loss, and total genes (Median Jaccard Index was 0.71, 0.77, and 0.67, respectively) (Fig. 4h).

## Usage Notes

The updated profiling files can be downloaded at Data Portal (http://fudan-pgx.3steps.cn/cdataportal/study/summary?id=FUSCC_BRCA_2022) for easy reuse. Meanwhile, online visualization exploration is made available in this portal, and overall statistical results of multi-omics data with clinical information are provided in the form of interactive charts. In addition, users can also query specific samples or genes according to their interests, and the results displayed in the portal will timely change.

## Supplementary information

Table S1.

Table S2.

Table S3.

Table S4.

Table S5.

Table S6.

## Data Availability

The pipeline applications we provided contain all the processes described by Workflow Description Language (WDL, https://github.com/openwdl/wdl) before R analyses. Please note that the parameters in them are fixed, and the sample files can be processed by direct invocation. The upstream analysis of WES and RNAseq is available at the GitHub repository (https://github.com/fudan-tnbc). The dockers used in the upstream analysis can be obtained in the Docker Hub (https://hub.docker.com/u/chenqingwang). The code for pre-processing the upstream result data and drawing the figures can be found in the GitHub repository (https://github.com/fudan-tnbc/TNBC-Multiomics). Quality control metrics for all data were collected in the metadata tables and visualized using R v4.1.2 (https://cran.r-project.org/, R development core team).
